# Neutrophils promote venular thrombosis by shaping the rheological environment for platelet aggregation

**DOI:** 10.1038/s41598-019-52041-8

**Published:** 2019-11-04

**Authors:** Daniel Puhr-Westerheide, Severin J. Schink, Matthias Fabritius, Laura Mittmann, Maximilian E. T. Hessenauer, Joachim Pircher, Gabriele Zuchtriegel, Bernd Uhl, Martin Holzer, Steffen Massberg, Fritz Krombach, Christoph A. Reichel

**Affiliations:** 1 Walter Brendel Centre of Experimental Medicine, Klinikum der Universität München, Ludwig-Maximilians-Universität München, Munich, Germany; 2 Department of Radiology, University Hospital, Ludwig-Maximilians-Universität München, Munich, Germany; 3 Department of Systems Biology, Harvard Medical School, Boston, Massachusetts USA; 4 Department of Plastic and Hand Surgery, Friedrich Alexander University Erlangen Nuernberg, Erlangen, Germany; 5 Department of Cardiology, Klinikum der Universität München, Ludwig-Maximilians-Universität München, Munich, Germany; 6 Department of Otorhinolaryngology, Klinikum der Universität München, Ludwig-Maximilians-Universität München, Munich, Germany

**Keywords:** Acute inflammation, Thrombosis, Platelets, Neutrophils

## Abstract

In advanced inflammatory disease, microvascular thrombosis leads to the interruption of blood supply and provokes ischemic tissue injury. Recently, intravascularly adherent leukocytes have been reported to shape the blood flow in their immediate vascular environment. Whether these rheological effects are relevant for microvascular thrombogenesis remains elusive. Employing multi-channel *in vivo* microscopy, analyses in microfluidic devices, and computational modeling, we identified a previously unanticipated role of leukocytes for microvascular clot formation in inflamed tissue. For this purpose, neutrophils adhere at distinct sites in the microvasculature where these immune cells effectively promote thrombosis by shaping the rheological environment for platelet aggregation. In contrast to larger (lower-shear) vessels, this process in high-shear microvessels does not require fibrin generation or extracellular trap formation, but involves GPIbα-vWF and CD40-CD40L-dependent platelet interactions. Conversely, interference with these cellular interactions substantially compromises microvascular clotting. Thus, leukocytes shape the rheological environment in the inflamed venular microvasculature for platelet aggregation thereby effectively promoting the formation of blood clots. Targeting this specific crosstalk between the immune system and the hemostatic system might be instrumental for the prevention and treatment of microvascular thromboembolic pathologies, which are inaccessible to invasive revascularization strategies.

## Introduction

Hemostasis is a fundamental biological process that prevents bleeding after vessel injury. This mechanism is not only beneficial for prohibiting hemorrhage, but also represents an instrument of the body to limit the systemic dissemination of pathogens from the site of injury by interrupting the blood flow in the underlying microvasculature^1–4^. Uncontrolled thrombus formation (e.g., in myocardial infarction, stroke, peripheral vascular disease, or sepsis), however, compromises blood supply and provokes ischemic tissue damage. Although the broad implementation of invasive techniques for the revascularization of larger vessels improved the treatment regiments for these pathologies^5–9^, therapeutic options for the dissolution of blood clots (remaining) in the inflamed microvessels of (post)ischemic tissue are still limited due to their inaccessibility to these interventional procedures.

Upon vascular damage, platelets immediately adhere to the injured vascular endothelium, form aggregates, and release vasoactive substances (e.g., serotonin, thromboxane) which causes vasoconstriction and a subsequent reduction of blood flow in the affected vessels (‘primary hemostasis’). In the course of these events, the plasmatic coagulation system is engaged to further stabilize the platelet thrombus (‘secondary hemostasis’)^1–4^. In addition to platelets and the coagulation system, leukocytes have recently been reported to collaborate for clot formation in larger vessels (as assessed in models of arterial injury and deep vein thrombosis) and hepatic sinusoids (during the clearance of circulating pathogens) in a process termed ‘immunothrombosis’: Under exposure to relatively low shear stress, neutrophils support thrombus growth by the release of extracellular traps (NETs; network of extracellular fibers containing DNA and other molecules^10^) which, in turn, activate components of the coagulation system and facilitate platelet aggregation^11–14^. Whether these elementary immune cell-driven events are relevant for the formation of thrombi in the microvasculature of inflamed tissue, however, is still unclear.

Various studies have demonstrated that interactions of leukocytes and platelets in the inflamed microvasculature are vital for proper immune cell responses^15–23^. Conversely, it has been shown that an inflammatory environment exacerbates the thrombotic phenotype of microvessels^24^. In this context, neutrophils have been reported to promote the clotting of the cerebral microvasculature in experimental sickle cell disease^25^. *In vivo* microscopy observations further suggest that such events require interactions of intravascularly adherent neutrophils with platelets that rely on thrombin, tissue factor, elastase, cathepsin G, and ATP/adenosine-dependent inhibition of tissue factor pathway inhibitor^26–28^.

In addition to these molecular mechanisms, rheological factors contribute to intravascular platelet adhesion and thrombus formation^29–32^. Interestingly, it has recently been reported that intravascularly adherent leukocytes shape the blood flow in their immediate vascular environment^33^. Consequently, we hypothesize that these distinct rheological effects arising from leukocytes recruited to the inner vessel wall of inflamed tissue propagate microvascular thrombus formation.

## Results

### Thrombus formation in the microvasculature of inflamed tissue

**Figure 1:**
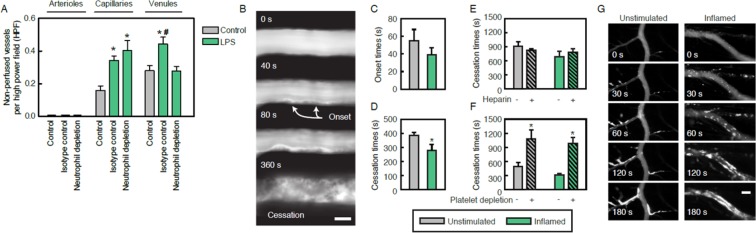
Spatio-temporal dynamics of thrombus formation in inflamed venular microvessels. Non-perfused arterioles, capillaries, and venules were quantified in the cremaster muscle of unstimulated control animals and in animals receiving an intrascrotal injection of LPS as well as intravenously neutrophil-depleting anti-Ly-6G mABs or isotype control Abs (**A**; mean ± SEM for n = 4 per group; *p < 0.05 vs. control, ^#^p < 0.05 vs. neutrophil depletion). Thrombus formation in postcapillary venules of the mouse cremaster muscle was induced by photochemical injury as detailed in *Methods*, representative *in vivo* fluorescence microscopy images of time-lapse video recordings are shown (**B**; scale bar: 20 μm, Video S1, 4). Panels show quantitative data for onset and cessation times in WT mice receiving a local, intrascrotal injection of PBS (‘unstimulated’) or LPS (‘inflamed’) (**C**,**D**; mean ± SEM for n = 9 per group; *p < 0.05 vs. unstimulated control) and undergoing treatment with heparin, platelet-depleting antibodies, or vehicle/isotype control antibodies (**E**,**F**; mean ± SEM for n = 3–4 per group; *p < 0.05 vs. vehicle/isotype control). Aggregation patterns of fluorescence-labeled platelets during thrombus formation in unstimulated or inflamed capillary and venular cremasteric vessels were visualized by multi-channel *in vivo* fluorescence microscopy as detailed in *Methods*, representative images of time lapse video recordings are shown (**G**; platelets in white, scale bar: 40 μm, Video S2, 3).

To investigate the mechanisms underlying microvascular thrombosis in inflamed tissue, we performed *in vivo* microscopy analyses in the mouse cremaster muscle. In unstimulated tissue, non-perfused microvessels were barely detected (Fig. 1A). Upon induction of inflammation (elicited by an intrascrotal injection of lipopolysaccharide (LPS)), however, the number of non-perfused capillaries and post-capillary venules was significantly increased, whereas arteriolar perfusion remained unaffected. This increase in numbers of non-perfused venules, but not in non-perfused capillaries, was significantly decreased in neutrophil-depleted animals.

To characterize the mechanisms underlying microvascular thrombus formation in inflamed tissue in more detail, we further enhanced the inflammatory status of selected parts of the tissue by applying a ‘second hit’ (photochemical injury; Video S1) which is supposed to injure endothelial cells (resulting in excessive release of von Willebrand factor (vWF) as observed under severe inflammatory conditions) without extensively destroying subendothelial structures (e.g., as opposed to models of ferric chloride-induced thrombosis^34,35^). This procedure allowed us to reproducibly visualize interactions of neutrophils, platelets, and endothelial cells in high spatio-temporal resolution during thrombus formation in a given vessel segment (see below). In *a priori* unstimulated venules, platelets were observed to adhere to the surface of microvascular endothelial cells immediately upon photochemical injury, whereas the complete occlusion of these microvessels by the growing thrombi occurred at later time points. Upon prestimulation of the cremaster muscle with LPS, however, cessation of blood flow was significantly accelerated and a slight trend towards an even faster onset of platelet adhesion was noted, although not reaching statistical significance (Fig. 1B–D). Importantly, thrombus formation in arterioles took a significant longer time than in venules while no significant differences between unstimulated and inflammatory conditions were observed (Fig. S1A,B).

### Role of platelets and the plasmatic coagulation for thrombus formation in the venular microvasculature of inflamed tissue

To identify the mechanisms underlying the accelerated thrombus formation in venular microvessels of inflamed tissue, we sought to evaluate the individual contributions of platelets and the plasmatic coagulation system to this process. Inhibition of fibrin generation with heparin (which also dismantles the NET scaffold and interferes with thrombin-mediated platelet activation) did not significantly alter the cessation times of blood flow upon photochemical injury in unstimulated or prestimulated venules (Fig. 1E). In contrast, antibody-mediated depletion of platelets (by > 90% as compared to controls; Table S1) substantially prolonged the average time to the cessation of blood flow upon photochemical injury in both unstimulated and inflamed venules (Fig. 1F).

To characterize the role of platelets for thrombus formation in venular microvessels in further detail, interactions of fluorescence-labeled platelets with endothelial cells were visualized by multi-channel *in vivo* fluorescence microscopy. In the *a priori* unstimulated cremaster muscle, discoid platelets began to adhere and aggregate upon photochemical injury predominantly in capillaries, from where the forming thrombi progressively grew into the lumen of venules. Having reached postcapillary venules, the single thrombi merged into large thrombi that finally occluded the venules together with their preceding capillary vessel segments. In striking contrast, discoid platelets were found to directly adhere at multiple sites on endothelial cells of prestimulated postcapillary venules instantly after photochemical injury thereby forming a venular thrombus. This process resulted in an accelerated cessation of blood flow in the capillary and venular vasculature (Fig. 1G; Video S2, 3, 4).

### Role of neutrophils for thrombus formation in venular microvessels of inflamed tissue

**Figure 2:**
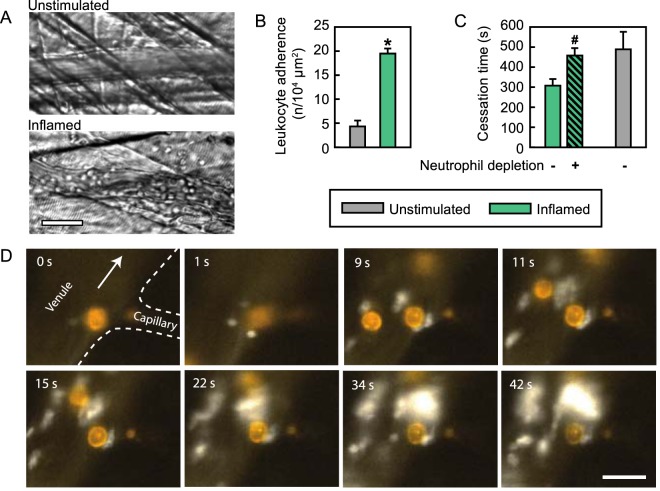
Role of neutrophils for thrombus formation in inflamed venular microvessels. Intravascular adherence of leukocytes was analyzed in postcapillary venules of the cremaster muscle of WT mice receiving an intrascrotal injection of PBS (‘unstimulated’, grey plot) or LPS (‘inflamed’, green plot) by RLOT *in vivo* microscopy as detailed in *Materials and Methods*, representative images are shown (**A**; scale bar: 40 μm). Panel (B) shows quantitative data for leukocyte intravascular adherence in venules (mean ± SEM for n = 4 per group; *p < 0.05 vs. unstimulated control). Thrombus formation in postcapillary venules of the cremaster muscle of WT mice was induced by photochemical injury as detailed in *Methods*. Panel (C) shows quantitative data for cessation times in WT mice undergoing intrascrotal stimulation with LPS after treatment with neutrophil-depleting or isotype control antibodies (mean ± SEM for n = 4 per group; *p < 0.05 vs. isotype control). Interactions of platelets (white) and neutrophils (orange) during thrombus formation in postcapillary venules of the cremaster muscle of WT mice induced by photochemical injury were visualized by multi-channel *in vivo* fluorescence microscopy as detailed in *Methods*, representative images are shown (**D**; arrow indicates flow direction, scale bar: 20 μm, Video S5, 6).

In a next step, we sought to evaluate the functional relevance of neutrophils for thrombus formation in the microvasculature of inflamed tissue. Performing *in vivo* microscopy in postcapillary venules of the unstimulated cremaster muscle, only few intravascularly adherent leukocytes were found. As expected, intravascular adherence and transmigration of leukocytes (>90% Ly-6G^+^ neutrophils) was significantly elevated in inflamed tissue as elicited by intrascrotal stimulation with LPS (Fig. 2A,B**)**, whereas leukocyte adherence in arterioles was virtually absent (0.4 ± 0.2/10^4^ μm^2^).

Antibody-mediated depletion of neutrophils (eliminating > 90% of neutrophils compared to controls; Table S2) completely reversed the acceleration of microvascular thrombus formation in LPS-stimulated cremasteric venules to the level of unstimulated venules (Fig. 2C) indicating that neutrophils significantly contribute to thrombus formation under these inflammatory conditions. *In vivo* immunostaining and multi-channel fluorescence microscopy revealed that platelets preferentially form aggregates in inflamed postcapillary venules at sites downstream of adherent or shortly adhered and subsequently detached neutrophils. From these multiple ‘hot spots’, platelet aggregates further grow and merge into one large thrombus finally occluding the entire venule (Fig. 2D; Video S5). Simultaneously, the number of intravascularly adherent neutrophils did not further increase upon thrombus formation in inflamed venules (Fig. S2). Noteworthy, we are not able to clearly answer the question whether the shortly adherent neutrophils ‘create’ appropriate vascular sites for platelets to accumulate or whether platelets and shortly adherent neutrophils simply adhere to similar vascular sites.

Previously, the role of neutrophils for venular thrombus formation has been analyzed upon intra-peritoneal administration of LPS, mimicking initial stages of a systemic inflammatory response^24^. In this study, depletion of neutrophils and inflammatory monocytes by anti-Gr-1 antibodies did not affect accelerated thrombus formation in cremasteric venules. We were able to reproduce these results in neutropenic animals (using neutrophil-selective anti-Ly-6G antibodies; Table S2) Fig. S3A,B. The missing effect of neutrophil/inflammatory monocyte depletion on venular thrombus formation here might be, at least in part, attributed to the low degree of local inflammation in the cremaster muscle under these specific experimental conditions (indicated by lacking intravascularly adherent leukocytes in cremasteric venules; Fig. S3C) as opposed to our findings under severe local inflammation (elicited by local, intra-scrotal injection of LPS) in which numerous neutrophils accumulated in the microvasculature (see above).

### Effect of physical characteristics of the inner microvessel surface on platelet deposition under flow

**Figure 3:**
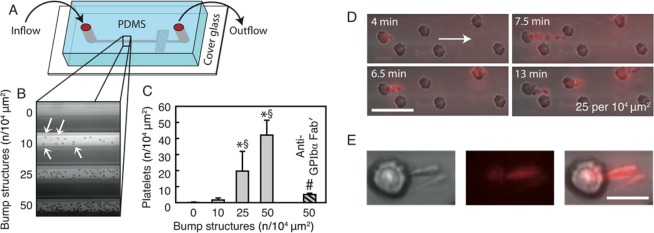
Rheological effects of adherent neutrophils on platelet aggregation under flow. Interactions of fluorescence-labeled platelets were analyzed *in vitro* in custom-made PDMS microfluidic devices as detailed in *Methods*, a schematic illustration of the device (**A**) and transillumination microscopy images of the flow channels (**B**) are shown (arrows indicate individual bump structures). Panel (C) shows quantitative data for the number of platelets adhering to the wall of flow channels exhibiting varying densities of bump structures (mimicking intravascularly adherent neutrophils) after 10 min of perfusion with mouse blood. In selected experiments, anti-GPIbα mAbs were added to the blood (mean ± SEM for n = 3–4 per group; *p < 0.05 vs. flat devices, ^§^p < 0.05 vs. lowest bump density, ^#^p < 0.05 vs. highest bump density). Interactions of fluorescence-labeled platelets with bump structures were analyzed *in vitro* in custom-made PDMS microfluidic devices by high-resolution microscopy as detailed in *Methods*, representative images are shown (**D**; scale bar: 20 μm). In panel (E; scale bar: 10 μm), formation of GPIbβ-positive tethers in platelets (red) adhering to bump structures is shown.

Based on our *in vivo* microscopy observations, we hypothesized that perturbations of the blood flow around intravascularly adherent neutrophils facilitate platelet capture and thrombus propagation. In order to separate physical effects from neutrophil-specific molecular interactions, we constructed a polydimethylsiloxane (PDMS) microfluidic device, which mimics the surface geometry of postcapillary venules and is uniformly thrombogenic (Fig. 3A). The device consists of four channels with different densities of ‘bump’ structures of the same material (Fig. 3B) mimicking intravascularly adherent neutrophils (8 µm in diameter, consistent with the protrusion of adherent neutrophils into the vessel lumen, Fig. S4A,B; up to 50 bumps per 10^4^ µm^2^ surface area). After 10 min of perfusion with mouse blood at physiological microvascular shear rates (1500 s^−1^), only few thrombocytes (fluorescence-labeled by an anti-GPIbβ mAb) were adherent in the channel with a ‘flat’ surface (mimicking unstimulated conditions). In channels with bumps (mimicking intravascularly adherent neutrophils in inflamed venules), however, the number of adherent platelets was dramatically increased as compared to the channel with a flat surface, more than 100-fold for the highest density tested.

Employing high-resolution time-lapse microscopy, we found that platelets were initially captured on the central region of a bump (Fig. 3D, Video S7; 4 min), from where these cellular blood components migrated on the bump to the obstacles’ downstream face (6.5 min). Having arrived in the wake of a bump, the first platelets began to form membranous (GPIbβ-positive) tethers anchoring to the bump (up to 15 μm in length; 7.5 min), which allowed the platelets to freely rope down in the bloodstream (Fig. 3E). These events, in turn, promoted the capturing of following platelets thereby facilitating aggregation of these blood components and promoting microvascular thrombus growth. Moreover, tethered platelets were also observed to adhere to downstream bumps thereby further increasing the number of growing microthrombi (Fig. 3D, 13 min). In line with previous observations^29,36^, platelets remained discoid in shape during the entire capture and aggregation process and adhered to the PDMS surface via deposited patches of vWF (but not strings of vWF, most probably due to ADAMTS13-dependent degradation of endothelially released vWF^32^; Fig. S5), which was abrogated when blood was incubated with anti-GPIbα Fab’ fragments before the onset of microfluidic experiments (Fig. 3C).

**Figure 4:**
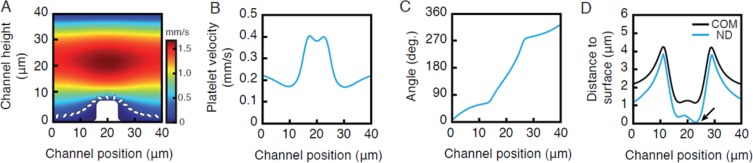
Theoretical analysis of platelet capture by intravascularly adherent neutrophils. Platelet motion in a flow channel exhibiting a bump structure was modeled along a longitudinal cross-section with a fluid dynamics simulation of an ellipsoid as detailed in *Methods*. Panel (A) shows the blood flow velocity profile (pseudocolors) and the motion kinetics of a platelet moving over the bump structure (white; assuming the absence of bimolecular interactions) in a flow channel. Platelet velocity (**B**) and its relative angle (**C**) during its movement over the bump structure are shown. Panel (D) shows that the acceleration of the platelet decreases the distance of the platelet’s center of mass to the bump surface (‘COM’). Additionally, a flipping motion brings one end of the platelet into close proximity to the bump surface, further reducing the nearest distance (‘ND’) and thus allowing biochemical binding (arrow).

For a better understanding of the hydrodynamic effect of bump structures on platelet recruitment, we employed a fluid dynamics simulation (Fig. 4A–D). We found that platelets are exposed to higher blood flow velocity gradients when bypassing the bump structure. This causes drag that induces a spinning and flipping motion in platelets, thereby eventually bringing the platelets’ surface in close contact to the bump’s surface allowing molecular interactions and subsequent adhesion.

### Molecular mechanisms underlying thrombus formation in the venular microvasculature of inflamed tissue

In a next step, we sought to elaborate the molecular basis of this neutrophil-driven acceleration of thrombus formation in the venular microvasculature of inflamed tissue. To avoid interference with inflammatory processes arising during the prestimulation period of the cremaster muscle (6 h stimulation with LPS), blocking antibodies directed against the target proteins were applied 10 min prior to the induction of photochemical injury and *in vivo* microscopy.

**Figure 5:**
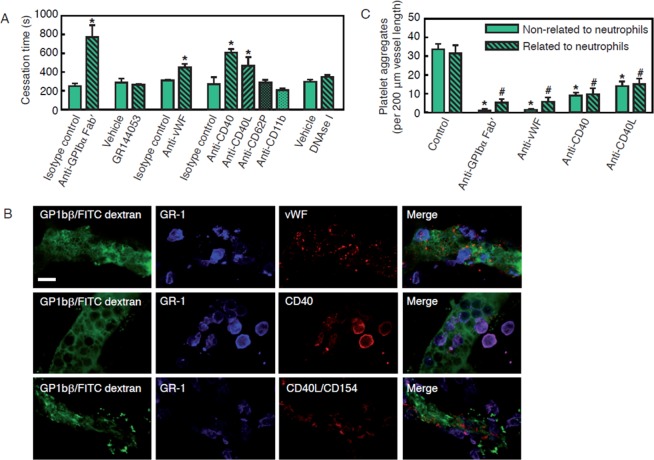
Molecular mechanisms underlying thrombus formation in inflamed venular microvessels. Thrombus formation in postcapillary venules of the cremaster muscle of WT mice was induced by photochemical injury as detailed in *Methods*. Panel (A) shows quantitative data for cessation times in animals undergoing intrascrotal stimulation with LPS and treatment with antibodies directed against P-selectin/CD62P, Mac-1/CD11b, vWF, GPIbα, CD40, or CD40L/CD154, with compound GR144053 (inhibitor of GPIIb/IIIa), with DNAse I (NET-degrading enzyme), or vehicle/isotype control antibodies (mean ± SEM for n = 4 per group; *p < 0.05 vs. vehicle/isotype control). Panel (B; scale bar: 10 μm) shows representative confocal microscopy images illustrating the spatial relationship of neutrophils/classical monocytes (Ly-6G/C; blue), platelets (GPIbβ; green) and vWF, CD40, or CD40L/CD154 (red) in thrombi occluding postcapillary venules of the inflamed cremaster muscle of WT mice. The spatial distribution of platelet interactions during thrombus formation in postcapillary venules of the cremaster muscle of WT mice induced by photochemical injury was analyzed by multi-channel *in vivo* fluorescence microscopy as detailed in *Methods*. Panel (C) shows quantitative data for animals undergoing intrascrotal stimulation with LPS and treatment with antibodies directed against vWF, GPIbα, CD40, CD40L/CD154, or isotype control antibodies (mean ± SEM for n = 3 per group; *p < 0.05 vs. baseline non-related to neutrophils, ^#^p < 0.05 vs. baseline related to neutrophils).

Inhibition of vWF or its major platelet receptor GPIbα, but not of its alternative platelet receptor GPIIb/IIIa, significantly prolonged the time to complete microvascular thrombus formation in inflamed venules as compared to isotype control/vehicle treated animals (Fig. 5A). Furthermore, blockade of CD40 or its ligand CD40L/CD154 lead to a significant prolongation of thrombus formation in the inflamed venular microvasculature as compared to controls. In contrast, blockade of P-selectin/CD62P (facilitating platelet- or leukocyte-endothelial cell and platelet-leukocyte interactions through leukocyte PSGL-1/CD162^16^) or Mac-1/CD11b (facilitating interactions of leukocytes with fibrin(ogen) or platelet GPIbα^37^) did not significantly alter thrombus formation in inflamed venular microvessels. Noteworthy, only few NETs were found in the inflamed cremaster muscle (Fig. S7) and disruption of these structures (that are able to activate platelets and the coagulation system in lower-shear vessels^11–14^) with DNase I did not affect microvascular thrombus formation (Fig. S6).

Employing *in vivo* immunostaining and multi-channel fluorescence microscopy as well as *ex vivo* confocal microscopy analyses, the role of vWF, GPIbα, CD40, and CD40L/CD154 for the spatial distribution of platelet interactions in the venular microvasculature was analyzed in a next step. Blockade of vWF (which is uniformly distributed throughout the entire thrombus) or of its platelet receptor GPIbα and – to a lesser degree – of CD40 (predominantly present on neutrophils and platelets) or its ligand CD40L/CD154 (predominantly present on platelets) significantly diminished the initial interactions of platelets in inflamed venules, but did not show any prevalence regarding the relation of platelets to adherent neutrophils (Fig. 5B,C).

### Systemic leukocyte counts, microhemodynamic parameters, and FITC dextran perfusion

To assure intergroup comparability, systemic leukocyte counts, microhemodynamic parameters including blood flow velocity, inner vessel diameter, and wall shear rate, as well as fluorescence intensities measured in analyzed FITC dextran-perfused vessel segments prior to induction of photochemical injury were determined in each experiment. No significant differences were detected among experimental groups (Table S3).

## Discussion

Microvascular thrombosis is a hallmark of advanced inflammatory disease which leads to the interruption of blood supply and provokes ischemic tissue injury^1,3,4^. In this context, immune cells have been observed to collaborate with platelets and the plasmatic coagulation system for the generation of blood clots, employing a complex molecular interplay. Recently, intravascularly adherent leukocytes have been reported to alter their surrounding rheological environment^33^. Whether these distinct effects of immune cells are relevant for microvascular thrombogenesis is still unknown.

To understand the mechanisms controlling microvascular thrombosis in inflamed tissue, we here sought to determine the basic principles underlying this commonly encountered process. In line with previous reports under early systemic inflammatory conditions^24^, thrombus formation in the microvasculature of inflamed tissue critically involved platelets, but did not require fibrin generation by the coagulation system (as opposed to thrombosis in arteries, veins, or liver sinusoids^1,2^). Most interestingly, inflammation dramatically accelerated thrombus formation in venules, but not in arterioles. A closer look on these microcirculatory events revealed that in unstimulated tissue thrombi originate from capillaries and progressively grow into the lumen of postcapillary venules ultimately occluding these vessel segments. In inflamed tissue, however, platelets directly accumulate at multiple sites in postcapillary venules thereby leading to an accelerated cessation of blood flow in the capillary and venular vasculature. Thus, inflammation changes the spatio-temporal interaction patterns of platelets in the microvasculature which effectively promotes microvascular clot formation. These experimental findings might contribute to the explanation of clinical observations suggesting inflammatory conditions as independent risk factor for thromboembolic events^38^.

Leukocyte recruitment to the site of inflammation is an integral part of the inflammatory response^39–43^. Whereas immune cells were initially thought to be dispensable for thrombus formation in the microvasculature (during initial stages of endotoxemia)^24^, recent observations implicated these immune cells in the generation of microvascular clots upon focal laser-injury of the *a priori* unstimulated vessel wall^27,28^. Since platelets primarily accumulated in postcapillary venules (representing the vessel segments where neutrophils arrest on the endothelium upon induction of inflammation^17^) during thrombus formation in inflamed tissue, we proposed that neutrophils critically support microvascular thrombosis under these pathological conditions. Consistent with our assumption, the acceleration of thrombus formation in the venular microvasculature was completely abolished in the absence of neutrophils clearly documenting that these cells drive inflammatory microvascular thrombosis. In this context, we found that platelets preferentially adhered at the vessel wall of postcapillary venules in areas downstream of adherent neutrophils during clot formation. At these ‘hot spots’, platelets immediately formed aggregates that gradually merged into a large thrombus occluding the entire vessel segment. Conversely, under inflammatory conditions lacking significant leukocyte responses (e.g., in peripheral tissues during initial stages of endotoxemia^24^), neutrophils are not involved in microvascular thrombogenesis. Hence, intravascularly adherent neutrophils recruit platelets to specific sites in the microvasculature of inflamed tissue from which thrombus formation can emerge. Our findings might thereby explain why neutropenia protects from thrombus formation in the cerebral microvasculature of mice suffering from experimental sickle cell disease (which is associated with leukocyte accumulation in microvessels)^25^.

With respect to these distinct interaction patterns of platelets in microvascular thrombosis, to the previously described streamlines around intravascularly adherent neutrophils^33^, and to reports on the shear-dependency of platelet interactions^30–32^, we further hypothesized that intravascularly adherent neutrophils promote platelet aggregation by perturbing the blood flow in the microvasculature. To analyze the contribution of such rheological effects of intravascularly adherent neutrophils to microvascular thrombogenesis, we constructed microfluidic devices consisting of flow channels with the size of postcapillary venules. In selected flow channels, the inner wall of these constructs exhibited ‘bump’ structures mimicking intravascularly adherent neutrophils. Upon perfusion with blood, we found that platelet deposition on the wall of these devices increased more than 100-fold when bumps were present. In this process, platelets adhered on the bump structure, migrated to its downstream face, and formed tethers anchoring to the obstacle. These membranous tethers (containing F-actin^44^), in turn, promoted the capture of following platelets ultimately mediating aggregation of these cellular blood components and facilitating thrombus growth. Theoretical simulations revealed that the initial platelet adhesion to bump structures is supported by hydrodynamic forces approximating platelets to the bump structure and additionally inducing a flipping motion^45^ in these cellular blood components when approaching the obstacle. This facilitates molecular binding of platelets and their subsequent arrest. Consequently, our data demonstrate for the first time that intravascularly adherent neutrophils promote microvascular thrombus formation by shaping the rheological environment for platelet aggregation.

Towards a more comprehensive understanding of the molecular mechanisms underlying neutrophil-driven thrombus formation in the microvasculature of inflamed tissue, we systematically screened a variety of candidate adhesion and signaling molecules for their functional relevance in this particular biological process^19,22,46,47^. In line with studies under early systemic inflammatory conditions^48–55^, clot formation in the inflamed venular microvasculature strictly required vWF (which is scattered throughout the thrombus) and its platelet counter receptor GPIbα, but not its alternative platelet receptor GPIIb/IIIa. In a more detailed analysis, we found that vWF mediated the initial intravascular interactions of platelets during thrombus formation. These vWF- and GPIbα-dependent events occurred irrespective of their relative localization to intravascularly adherent neutrophils. Blockade of other adhesion/signaling molecules facilitating interactions of platelets and neutrophils, including the β2 integrin CD11b/Mac-1 (leukocyte receptor for GPIbα^56,57^) or CD62P/P-selectin (endothelial and platelet ligand for leukocyte PSGL-1/CD162^16,52,58,59^), did not affect thrombus growth. Accordingly, blockade of GPIbα completely disrupted the attachment of discoid platelets to bump structures in our microfluidic channels, collectively suggesting that vWF serves as the ‘molecular glue’ for platelets during the onset of microvascular thrombus formation. Furthermore, we show that CD40 (present on adherent leukocytes and platelets) and its ligand CD40L (predominantly present on platelets) are also – but to a much lesser degree – involved in microvascular thrombosis in inflamed tissue, extending previously published observations^60^. Taken together, neutrophils play a critical role in the initiation of microvascular thrombus formation in inflamed tissue by facilitating GPIbα-vWF- and CD40-CD40L/CD154-depedent thrombus growth. To which extent physical rheological effects and neutrophil-specific molecular interactions mediate platelet accumulation in the inflamed venular microvasculature cannot clearly be stated.

In addition to adhesion and signaling molecules, extracellular traps released by neutrophils have recently been demonstrated to activate platelets and to bind components of the coagulation system thereby promoting thrombosis in lower-shear vessels such as liver sinusoids, arteries, and veins^11–13^. In line with recent observations^61^, however, NETs were dispensable for thrombus formation in the inflamed (high shear) microvasculature. These findings might be explained by the limited mechanical stability of complexes between DNA and other proteins (e.g., fibrin) under high shear stress^62^ collectively emphasizing neutrophil-driven thrombosis in this common type of microvasculature as a biological process distinct from the mechanisms triggering ‘immunothrombosis’ in vascular beds with lower shear rates.

Whereas invasive intervention techniques for the removal of thrombi from arteries and veins revolutionized the treatment of thromboembolic events^6–8^, therapeutic options for the dissolution of blood clots (remaining) in smaller vessel segments, are still limited due to their inaccessibility to interventional procedures. Consequently, our findings urgently suggest the inclusion of neutrophils as targets in treatment regimens for microvascular thrombosis.

**Figure 6:**
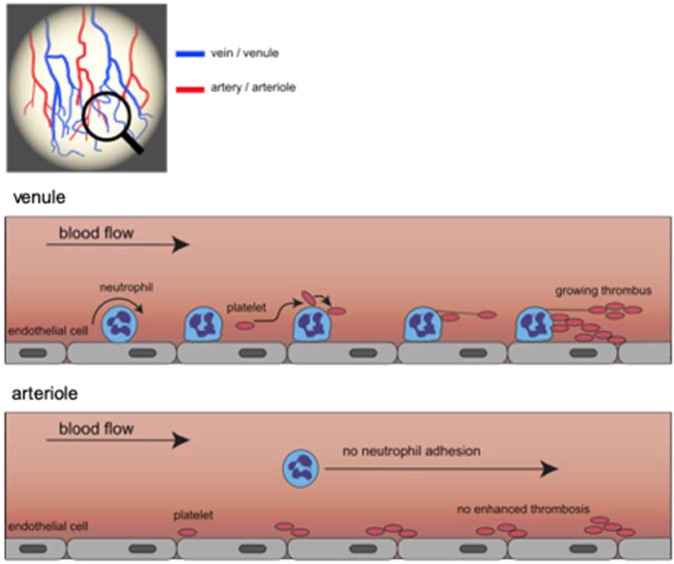
Graphical synopsis. In injured tissue, circulating neutrophils (blue) are recruited to postcapillary venules, where they arrest on microvascular endothelial cells. Supported by hydrodynamic forces, circulating platelets (dark red) get in close contact with neutrophils and the endothelium. This enables platelets to form tethers anchoring to adherent neutrophils and endothelial cells, thus accelerating thrombus formation. In arterioles, neutrophils are not recruited to the vessel wall and platelet thrombus formation is not facilitated by these immune cells.

In conclusion, our experimental data uncover previously unrecognized functional properties of neutrophils in inflamed tissue that endow these immune cells with the ability to promote thrombosis by shaping the rheological environment in the venular microvasculature for platelet aggregation (Fig. 6). Targeting this distinct, leukocyte-directed interplay between the immune system and the hemostatic system might be instrumental to prevent microvascular clotting in advanced stages of inflammatory pathologies.

## Methods

### Animals

For our experiments, male C57BL/6 J mice (ageing 8–12 weeks; Charles River (Sulzfeld, Germany) were used. Animals had free access to food and water and were housed under conventional conditions. All experiments were conducted according to the German legislation for the protection of animals with approval of the local government authorities (‘Regierung von Oberbayern’).

### Reagents

Lipopolysaccharide (LPS, Escherichia coli O111:B4; 10 ng intrascrotally (i.s.) or 1 mg intraperitoneally (i.p.); Sigma-Aldrich, Schnelldorf, Germany) was used to induce inflammation of the cremaster muscle or endotoxemia. Anti-GPIbα/CD42b monoclonal antibody (mAb; clone Xia.B2; 50 μg i.v.; 48 h, 24 h, and 6 h prior to induction of inflammation; emfret Analytics, Eibelstadt, Germany) was used for the depletion of platelets^17^. Anti-Ly-6G mAb (clone 1A8; 150 μg intravenously (i.v.); 24 h and 6 h prior to induction of inflammation; BD Biosciences, San Jose, CA, USA) was used for the depletion of neutrophils^63^. Microvascular thrombus formation was analyzed upon administration of heparin (500 IU intraarterially (i.a.); 10 min prior to induction of thrombosis; ratiopharm GmbH, Ulm, Germany), GPIbα blocking antibody Fab’ fragments (Xia.B2; 2.5 mg/kg, p0p/B, i.a.; 10 min prior to induction of thrombosis; emfret Analytics), a non-peptic GPIIb/IIIa inhibitor (GR144053 trihydrochloride; 10 mg/kg i.a.; 10 min prior to induction of thrombosis; R&D Systems, Lille, France), anti-vWF polyclonal Ab (100 μg in 100 μl PBS i.a.; 10 min prior to induction of thrombosis; Dako Deutschland GmbH, Hamburg, Germany), anti-CD40 mAb (clone 1C10; 50 µg in 100 µl PBS i.a.; 10 min prior to induction of thrombosis; eBiosciences, San Diego, CA, USA), anti-CD40L/CD154 mAb (clone MR1, 50 µg in 100 µl PBS i.a.; 10 min prior to induction of thrombosis; eBiosciences), anti-CD62P mAb (clone RB40.34; 50 μg in 100 μl PBS i.a.; 10 min prior to induction of thrombosis; BD Bioscience), anti-CD11b mAb (clone M1/70; 50 µg in 150 µl saline; BioLegend, San Diego, CA, USA), DNAse I (RNAse free; 100 U per mouse according to previous protocols^11,12^ and 1000 U per mouse according to previous studies^64^); i.a.; 4 h and 10 min prior to induction of thrombosis; Thermo Fisher Scientific, MA, USA), or appropriate vehicle/control antibodies.

Neutrophils were visualized by *in vivo* microscopy using an anti-Ly6G mAb (clone 1A8; PE-labeled; 5 μg in 100 μl PBS i.a.; BD Biosciences or clone AP-MAB0866; DyLight 550-labeld, 10 µg in 100 µl PBS i.a.; Novus Biologicals, Littleton, CO, USA). Platelets were visualized *in vivo* and *in vitro* by an anti-GPIbβ derivate (X-649; DyLight 649-labeled; 5 μg in 100 μl PBS i.a.; emfret Analytics). In microfluidic devices, vWF was visualized by an anti-vWF polyclonal Ab (100 μg per ml blood; Dako Deutschland GmbH, Hamburg, Germany) conjugated with a secondary anti-rabbit IgG antibody (green; Alexa Fluor 488 chicken anti-rabbit IgG; Invitrogen, Thermo Fisher Scientific, Rockford, IL). Please see the Major Resource Table in the Supplemental Material.

### *In vivo* microscopy on the mouse cremaster muscle

#### Surgical procedure

The surgical preparation of the mouse cremaster muscle was performed as originally described by Baez^65^ with minor modifications^66^. Briefly, mice were anesthetized using a ketamine/xylazine mixture (100 mg/kg ketamine and 10 mg/kg xylazine), administered by intraperitoneal (i.p.) injection. The left femoral artery was cannulated in a retrograde manner for administration of microspheres, antibodies, or inhibitors. The right cremaster muscle was exposed through a ventral incision of the scrotum. The muscle was opened ventrally in a relatively avascular zone, using careful electrocautery to stop any bleeding, and spread over the pedestal of a custom-made microscopy stage. Epididymis and testicle were detached from the cremaster muscle and placed into the abdominal cavity. Throughout the procedure as well as after surgical preparation during *in vivo* microscopy, the muscle was superfused with warm buffered saline.

#### Photochemical injury

Microvascular thrombus formation was induced by photochemical injury as described before^52^. For this purpose, animals received an i.a. injection of a FITC-dextran solution (150 kDa, 2,5%, 6 ml/kg bodyweight, Sigma-Aldrich). 5 min later, the vessel of interest (300 μm in length) was exposed to continuous epi-illumination using the FITC filter cube and appropriate illumination (λ = 488 nm). The field of illumination covers the entire field of view under investigation (as shown in figures and videos; in selected experiments all types of microvessels were epi-illuminated: Video S2, S3, S6). To assure intergroup comparability, the mean fluorescence intensity was determined in each of the analyzed vessels immediately after onset of light exposure.

#### *In vivo* microscopy

The setup for *in vivo* microscopy was centered around an Olympus BX 50 upright microscope (Olympus Microscopy, Hamburg, Germany), equipped for stroboscopic fluorescence epi-illumination microscopy as described before^67^. Briefly, light from a 75-W xenon source was narrowed to a near-monochromatic beam of a wavelength of 700 nm by a galvanometric scanner (Polychrome II, TILL Photonics, Graefelfing, Germany) and directed onto the specimen via a FITC filter cube equipped with dichroic and emission filters (DCLP 500, LP515, Olympus). Microscopy images were obtained with Olympus water immersion lenses [60×/numerical aperture (NA) 0.9, 20×/NA 0.5, and 10×/NA 0.3] and recorded with an analog black-and-white charge-coupled device (CCD) video camera (Cohu 4920, Cohu, San Diego, CA, USA) and an analog video recorder (AG-7350-E, Panasonic, Tokyo, Japan).

Selected experiments (Fig. S4) were obtained using a Axio Scope.A1 microscope equipped with a Colibiri 2 LED light source (Carl Zeiss Microscopy GmbH, Göttingen, Germany) and a QUAD filter set (QUAD DAPI (4′,6-diamidino-2-phenylindole)/FITC (fluorescein isothiocyanate)/Cy3/Cy5 sbx HC Filterset; AHF Analysentechnik AG, Tübingen, Germany) for fluorescence epi-illumination microscopy as described before^17^. Microscopy images were obtained with 20x and 40x water immersion lenses (0.5 NA, Zeiss MicroImaging GmbH).

To visualize the behavior of leukocytes and platelets during microvascular thrombus formation, selected experiments were performed using an *in vivo* microscopy setup centered around an AxioTech-Vario 100 Microscope (Zeiss MicroImaging GmbH, Goettingen, Germany), equipped with a Colibiri LED light source (Zeiss MicroImaging GmbH) for fluorescence epi-illumination microscopy as described before^68^. Briefly, light was directed onto the specimen via filter set 62 HE (Zeiss MicroImaging GmbH) fitted with dichroic and emission filters [TFT 495 + 610 (HE); TBP 527 + LP615 (HE)]. Microscopy images were obtained with an AxioCam Hsm digital camera using a 20x and 40x water immersion lenses (0.5 NA, Zeiss MicroImaging Gmbh). The images were processed with AxioVision 4.6 software (Zeiss MicroImaging GmbH).

#### Quantification of leukocyte kinetics and microhemodynamic parameters

*In vivo* microscopy records were analyzed offline using ImageJ (National Institutes of Health, Bethesda, MD) and Cap-Image (Dr. Zeintl, Heidelberg, Germany) image analysis software as described before^69^. Briefly, the onset of thrombus was defined as the first observation of endothelially adherent platelets during thrombus formation. Cessation of thrombus formation was defined as the complete occlusion of the vessel segment with flow cessation for > 60 s. The cut off time for thrombus formation in venules was 1500 s, in arterioles 2700 s. Firmly adherent leukocytes were determined as those resting in the associated blood flow for 30 s and related to the luminal surface per 100 μm vessel length. Transmigrated leukocytes were counted in regions of interest (ROI), covering 75 μm on both sides of a vessel over 100 μm vessel length. Non-perfused micorvessels in controls and LPS treated animals were quantified by counting occluded arterioles, capillaries and venules per high power field (HPF) with 175 HPF per animal this being equivalent to the total quantifiable area of an exteriorized cremaster muscle. By measuring the distance between several images of one fluorescent bead under stroboscopic illumination, centerline blood flow velocity was determined. From measured vessel diameters and centerline blood flow velocity, apparent wall shear rates were calculated, assuming a parabolic flow velocity profile over the vessel cross-section.

#### Experimental protocols and groups

In a first set of experiments, perfusion of arterioles, capillaries, and venules was analyzed in 15 high power fields (HPF) in a central area of the spread-out cremaster muscle of unstimulated WT mice and in WT mice undergoing four hours of intrascrotal stimulation with LPS as well as receiving an intra-venous application of neutrophil-depleting anti-Ly-6G mAb or isotype control Ab (n = 4 per group). In further experiments, thrombus formation in three randomly chosen postcapillary venules (diameter: 30–50 µm) and one arteriole (diameter: 20–35 µm) was analyzed upon photochemical injury 6 h after intra-scrotal injection of PBS or LPS (n = 9 per group). In separate experiments, the effect of heparin (administration 10 min prior to photochemical injury), platelet depletion, or neutrophil depletion on microvascular thrombus formation was analyzed 6 h after intra-scrotal or intra-peritoneal injection of PBS or LPS (n = 3–4 per group). In further experiments, the role of various adhesion/signaling molecules potentially mediating interactions of endothelial cells, leukocytes, and/or platelets for venular thrombus formation was analyzed upon application of blocking mAb or inhibitors (administration 10 min prior to photochemical injury) 6 h after i.s. injection of LPS (n = 4 per group). After *in vivo* microscopy, blood samples were collected by puncturing the inferior *vena cava* for the determination of systemic leukocyte and platelet counts using a ProCyte Dx cell counter (IDEXX Laboratories, Westbrook, ME, USA). Anesthetized animals were then killed by bleeding to death.

### Microfluidic experiments

#### Construction of microfluidic devices

Microfluidic devices were fabricated by molding silicone elastomer (PDMS Slygard 184, Dow Corning; Midland, MI, USA) to master molds constructed by photolithography on silicon wafers. The mold consisted of two layers of SU-8 photoresist (MicroChem Corp., Westborough, MA, USA), created by two superimposed patterns. A 32 µm thick layer of the channel layout was superimposed with an 8 µm thick layer of the channel layout with cut-outs of ‘bump structures’, yielding a negative mold with 40 µm thick channels and 8 µm thick bumps. The bump diameter was chosen to be 8 µm, resulting in cylindrical bumps with blunt edges due to photolithography limitation. The channel width was chosen to be 40 µm, resulting in a quadratic 40 µm × 40 µm cross-section. The channel layout consisted of four channels of different bump densities (0, 10, 25, and 50 bumps per 10^4^ µm^2^), fed from the same source and leading into the same sink. Bumps were randomly placed at non-overlapping positions throughout the channel using a standard MATLAB random number generator routine. A serpentine (40 µm × 40 µm × 16 mm) between channels and sink was designed as hydrodynamic resistance to reduce the flow rates to physiological conditions. The PDMS was mixed according to the manufacturer’s instructions, poured onto the wafer and cured for one hour at 80 °C. The cured PDMS was separated from the wafer and inlets and outlets were punched. The chip was then cleaned, exposed for 30 s to oxygen plasma (Diener electronics GmbH + Co. KG, Ebhausen, Germany) and irreversibly bond to a microscope slide.

#### Fluorescence microscopy

Microscopy was performed using a Nikon Ti-E inverted microscope system with automated stage and shutters controlled by NIS-Elements (Nikon, Tokio, Japan). Five positions spanning a total of 40 µm × 830 µm were recorded in each of the four channels every minute using a CFI P-Apo DM 100x objective and a SCMOS camera (Zyla-5.5, Andor, Belfast, Northern Ireland). Platelets were labeled with anti-GPIbβ derivate (X-649; emfret Analytics, Germany) and fluorescence emission was recorded.

#### Experimental protocol

Microfluidic chips were flooded and flushed with phosphate-buffered saline (PBS) before microfluidic flow experiments. A reservoir of heparinized mouse whole blood was connected to the microfluidic chip by silicone tubing and directed into the inflow of the device. Four channels with different densities of bump structures (as described above) were simultaneously perfused with whole blood containing fluorescence-labeled platelets. Upon application of fluorescence-labeled microspheres, the distance between several images of one fluorescent bead under stroboscopic illumination was measured allowing to determine the centerline blood flow velocity in the flow channels and to calculate the apparent wall shear rates as described above. The flow velocity was controlled by modulating the hydrostatic pressure, ultimately adjusting a shear rate of 1500 s^−1^ in the flow channels (corresponding to the shear rates present in the analyzed postcapillary venules; Table S3). After 10 minutes of perfusion, the number of adherent platelets was counted. In separate experiments, blood was diluted 10-fold with PBS for time-lapse high-resolution microscopy in order to avoid that the high density of cells obstructed the microscopy light passing through the cross-section of the channel. During perfusion, time-lapse microscopy was performed on the four channels to visualize capture, adhesion, and aggregation of platelets. In additional experiments, vWF deposition was visualized after 10 min of perfusion of PDMS channels with blood containing differentially fluorescence-labeled anti-vWF antibodies and platelets.

#### Fluid dynamics simulation

The fluid was simulated in a geometry representing a venule of 40 µm diameter under physiological flow conditions. We simulate a longitudinal cross section of the venule (2D, 160 µm × 40 µm), due to the observed adherence over the top of the bump structure, using commercial finite element simulation tools (COMSOL Multiphysics, Stockholm, Sweden). Due to the low Reynolds numbers found in the microvasculature, we assumed laminar flow. The platelet was considered a discrete, inelastic object that due to its small size does not alter the fluid flow in its vicinity. This allows for calculation of the fluid flow first, and afterwards using the solution to solve the translational and rotational movement of the platelet. Translation was calculated assuming Stokes drag, using the fluid velocity at the center of mass of the platelet. Rotation was induced by choosing the rotational velocity such that the average torque along the surface of the platelet was zero. Both translational and rotational movements in this approximation do not alter the fluid flow and were calculated using a custom-written MATLAB (Mathworks, Natick, MA, USA) script.

#### Confocal microscopy

For the analysis of CD40, CD40L/CD154, or vWF expression in microvascular thrombi, mouse cremaster muscles were prepared (6 h after i.s. injection of 10 ng LPS diluted in 400 µl PBS) and microvascular thrombus formation was induced by photochemical injury as described above. Excised cremaster muscles were fixed in 4% paraformaldehyde as described before^70^. Tissues were then blocked and permeabilized in PBS, supplemented with 10% goat serum (Sigma Aldrich) and 0.5% Triton X-100 (Sigma Aldrich). After incubation at 4 °C for 12 h with antibodies directed against GPIbβ derivate (X-649; DyLight 488-labeled; emfret Analytics), Ly6G/C (GR-1; Alexa Fluor 647-labeled; Invitrogen), and CD40 (rat anti-mouse; eBiosciences), CD40L/CD154 (rat anti-mouse; BD Bioscience), or vWF (rabbit polyclonal; abcam), tissues were incubated were incubated with an Alexa Fluor 546-linked goat anti-rat antibody or an Alexa Fluor 546-linked goat anti-rabbit antibody for 180 min at room temperature. For the visualization of NETs, cremaster muscle tissue whole mounts were incubated overnight at 4 °C with rabbit polyclonal anti-mouse histone H3 (citrulline R2 + R8 + R17) antibodies and rat monoclonal anti-mouse Ly6G (Abcam) antibodies. Secondary Alexa488- and Alexa594-conjugated antibodies (Invitrogen) were subsequently employed. DNA was stained with 1 µg/mL DAPI (Invitrogen).

Immunostained tissues were mounted in PermaFluor (Beckmann Coulter, Fullerton, CA) on glass slides. Confocal z-stacks typically covering approximately 30 µm (z-spacing 1.0 µm) were acquired using a Leica SP5 confocal laser-scanning microscopy (Leica Microsystems, Wetzlar, Germany) with an oil-immersion lens (Leica; 40×; NA 1.40).

#### Statistics

Data analysis was performed as described before^71^ with a statistical software package (SigmaStat for Windows; Jandel Scientific). The ‘t-test’ (two groups) or the ‘One-way ANOVA test’ followed by the ‘Dunnett test’ (>two groups, versus control) or followed by the ‘Student-Newman-Keuls method’ (>two groups, versus all other groups) was used for the estimation of stochastic probability in intergroup comparisons. Mean values and SEM are given. P values < 0.05 were considered significant.

## Supplementary information

Supplementary information

Video S1

Video S2

Video S3

Video S4

Video S5

Video S6

Video S7
