# Supplementary material for: Neutrophils promote venular thrombosis by shaping the rheological environment for platelet aggregation
Source: Sci Rep. 2019 Nov 4;9:15932. doi: 10.1038/s41598-019-52041-8 (PMC6828708; doi:10.1038/s41598-019-52041-8)
Supplement: Supplementary file 1 — Supplementary information [file 41598_2019_52041_MOESM1_ESM.pdf]

SUPPLEMENTAL MATERIAL

Neutrophils promote venular thrombosis by shaping the rheological environment for platelet aggregation

Daniel Puhr-Westerheide [1,2], Severin J. Schink [3], Matthias Fabritius [1,2], Laura Mittmann[1,6], Maximilian E. T. Hessenauer [1,4], Joachim Pircher [1,5], Gabriele Zuchtriegel[1,6], Bernd Uhl [1,6], Martin Holzer [1,6], Steffen Massberg [5], Fritz Krombach [1], and Christoph A. Reichel [1,6*]

[1] Walter Brendel Centre of Experimental Medicine, Klinikum der Universität München, Ludwig-Maximilians-Universität München, Munich, Germany

[2] Department of Radiology, University Hospital, Ludwig-Maximilians-Universität München, Munich, Germany

[3] Department of Systems Biology, Harvard Medical School, Boston, Massachusetts, USA

[4] Department of Plastic and Hand Surgery, Friedrich Alexander University Erlangen Nuernberg, Erlangen, Germany

[5] Department of Cardiology, Klinikum der Universität München, Ludwig-Maximilians-Universität München, Munich, Germany

[6] Department of Otorhinolaryngology, Klinikum der Universität München, Ludwig-Maximilians-Universität München, Munich, Germany

Supplemental figures

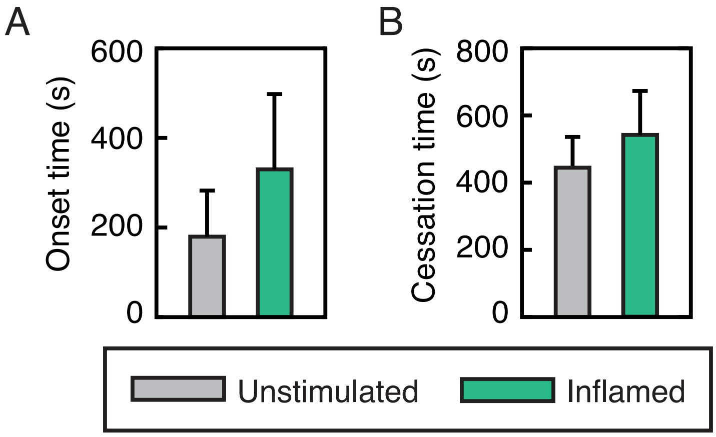

Figure S1. Thrombus formation in arterioles at the primary site of inflammation. Thrombus formation in arterioles of the cremaster muscle of WT mice was induced by photochemical injury as detailed in *Methods*. Panels show quantitative data for onset (**A**) and cessation (**B**) times in WT mice after intrascrotal injection of PBS or LPS (mean±SEM for n=4 per group).

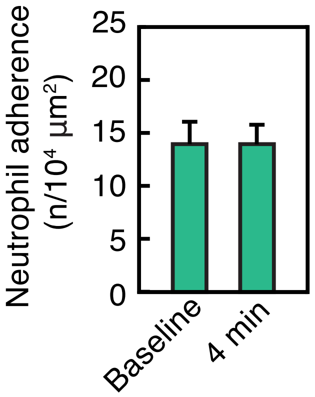

Figure S2. Neutrophil adhesion during venular thrombosis at the primary site of inflammation. Adhesion of fluorescence-labeled neutrophils was measured in the inflamed venular microvasculature prior ('Baseline') and 4 min after the onset of thrombus formation by photochemical injury as detailed in *Methods*. The panel shows quantitative results for the number of neutrophils being adherent to the vessel wall (mean±SEM for n=4 per group).

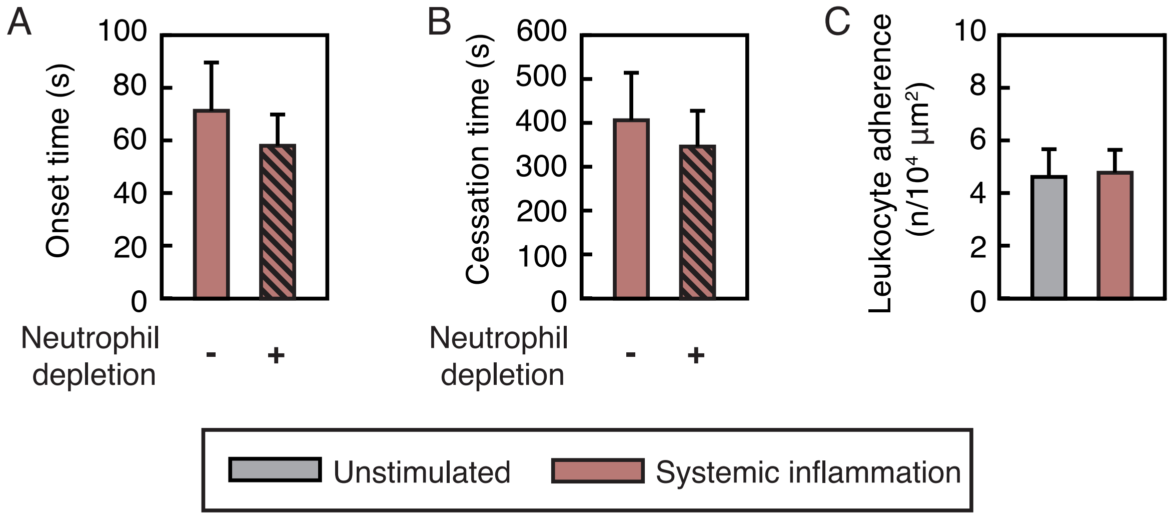

Figure S3. Microvascular thrombosis in early stages of endotoxemia. Thrombus formation in venules of the cremaster muscle of WT mice was induced by photochemical injury as detailed in *Methods*. Panels show quantitative data for onset (**A**) and cessation (**B**) times of thrombus formation 6 h after intra-peritoneal injection of LPS as well as the number of intravascularly adherent leukocytes 6 h after intra-peritoneal injection of PBS or LPS (mean±SEM for n=4 per group).

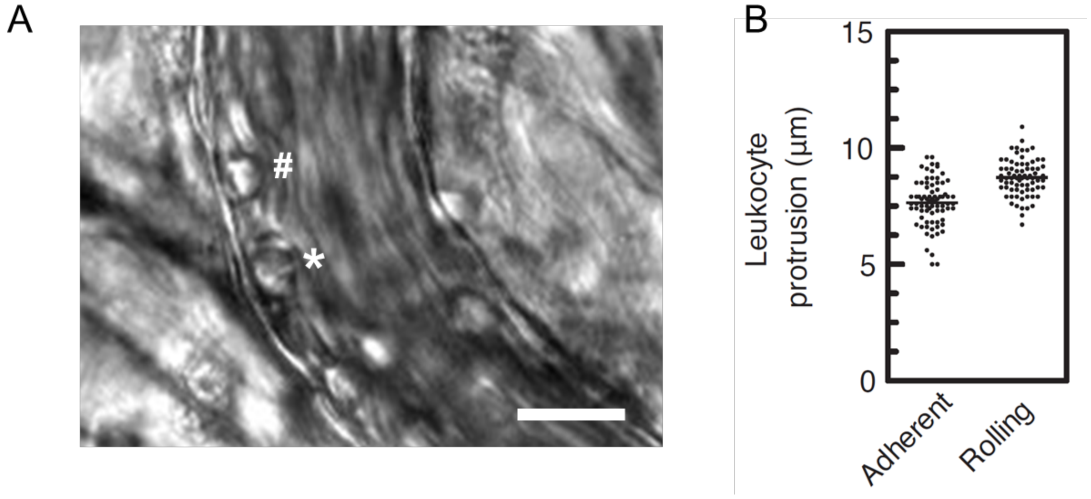

Figure S4. Leukocyte protrusion into the vessel lumen in inflamed venules. The protrusion of adherent and rolling leukocytes into the vessel lumen was analyzed by *in vivo* transillumination microscopy, a representative image is shown (**A**; # adherent leukocyte; * rolling leukocyte; scale bar: 20 µm). Panel (**B**) shows quantitative data for adherent and rolling leukocytes (**B**; n=75-77 cells per group).

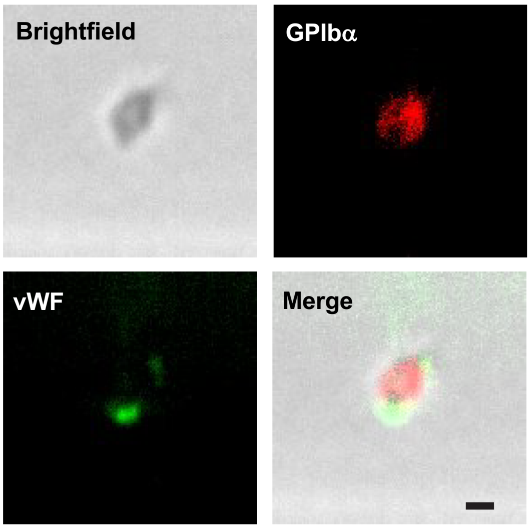

Figure S5. Platelet binding to PDMS via vWF. Blood containing anti-vWF antibodies (green) and fluorescence-labeled platelets (GPIbβ; red) was perfused through microfluidic PDMS channels under high shear (1500 s^{-1}) as detailed in *Methods*, representative fluorescence microscopy images are shown (scale bar: 1 μm).

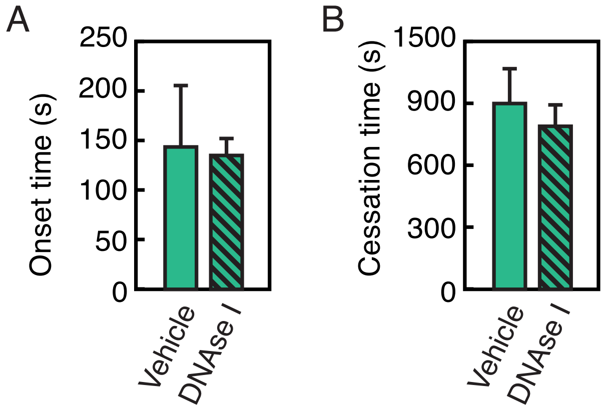

Figure S6. Microvascular thrombosis in inflamed tissue after treatment with DNAse I (1000 U). Thrombus formation in venules of the cremaster muscle of WT mice was induced by photochemical injury as detailed in *Methods*. Panels show quantitative data for onset (**A**) and cessation (**B**) times of thrombus formation 6 h after intra-scrotal injection of LPS in animals treated with DNAse I (1000 IE; for disrupture of NETosis) or vehicle 10 min prior to photochemical injury (mean±SEM for n=3 per group).

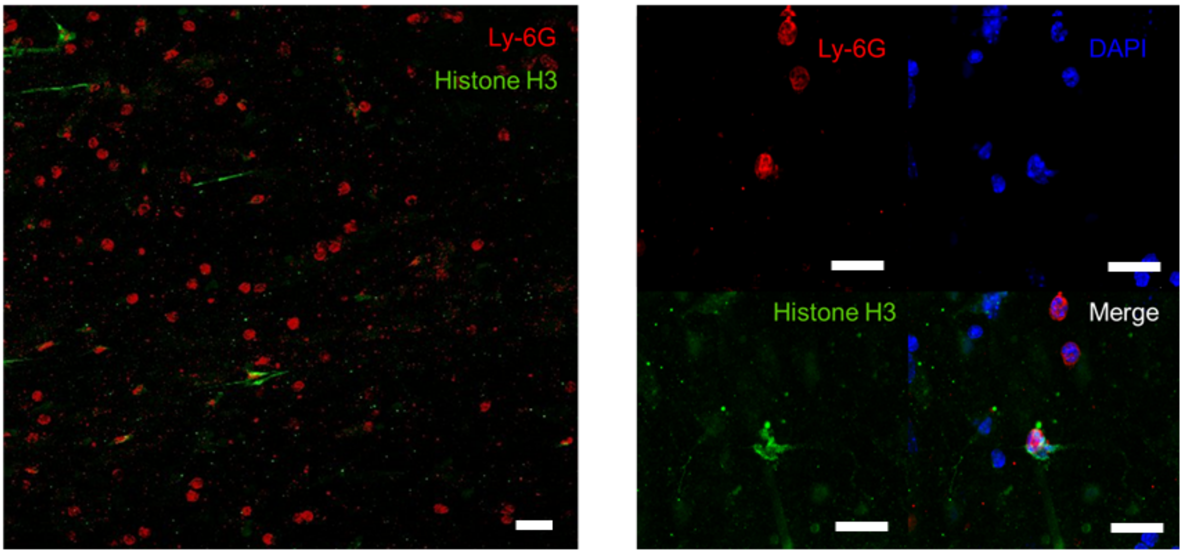

Figure S7. NETosis in the inflamed cremaster muscle. Formation of extracellular traps (histone H3; green) of neutrophils (Ly-6G-positive; red) was analyzed in tissue whole mounts of the mouse cremaster muscle upon stimulation with LPS by (immuno)staining and confocal microscopy, representative images are shown (scale bars: 20 µm; DAPI nuclear staining: blue).

Supplemental tables

Table S1. Platelet depletion. Systemic counts of platelets and white blood cells (WBC) were determined in the peripheral blood of WT mice treated with platelet-depleting or isotype control antibodies as detailed in *Methods*. Quantitative results are shown (mean±SEM for n=4 per group).

**Stimulus**	**Treatment**	**Platelets** (x 10^3 /µl)	**WBC** (x 10^3 /µl)
Unstimulated	Isotype	730±47	4.97±0.26
Unstimulated	Anti-GPIbα	42±11	4.28±0.18
LPS 10 ng i.s.	Isotype	608±55	5.29±0.56
LPS 10 ng i.s.	Anti-GPIbα	59±18	4.14±0.99

Table S2. Neutrophil depletion. Systemic counts of total white blood cells (WBC) and neutrophils were determined in the peripheral blood of WT mice treated with neutrophil-depleting or isotype control antibodies as detailed in *Methods*. Quantitative results are shown (mean±SEM for n=3-4 per group).

**Stimulus**	**Treatment**	**WBC** (x 10^3 /µl)	**Neutrophils** % WBC	**Neutrophils** (x 10^3 /µl)
Unstimulated	Isotype	4.97±0.26	25.80±2.00	1.3±0.1
LPS 10 ng i.s.	Isotype	5.29±0.56	20.80±2.90	1.1±0.1
LPS 10 ng i.s.	Anti-Ly6G	4.20±0.38	2.90±0.41	0.1±0.1
LPS 1 mg i.p.	Isotype	4.74±1.88	51.1±10.5	2.6±0.1
LPS 1 mg i.p.	Anti-Ly6G	3.11±1.04	9.7±4.2	0.3±0.0

Table S3. Systemic leukocyte counts, microhemodynamic parameters, and FITC dextran perfusion. Systemic leukocyte counts, microhemodynamic parameters, including inner vessel diameter, blood flow velocity, and wall shear rate, as well as fluorescence intensities measured in analyzed FITC dextran-perfused vessel segments prior to induction of photochemical injury were obtained as detailed in *Methods* (mean±SEM for n=3–9 per group).

Treatment	Diameter (µm)	Vmean (mm s^{-1})	Shear rate (s^{-1})	Fluorescence intensity (MFI)
None	38.8±0.7	1.5±0.1	1490.6±55.5	2077.0±123.2
None	38.2±0.7	1.3±0.1	1348.1±55.4	2064.9±103.9
Vehicle	38.6±0.3	1.4±0.1	1461.9±125.5	2063.4±50.3
Heparin	36.8±0.5	1.5±0.1	1575.7±56.6	2128.3±40.5
Vehicle	37.9±0.2	1.4±0.1	1502.0±112.8	2044.9±55.5
Heparin	36.6±0.6	1.3±0.1	1425.4±88.4	1962.5±39.2
Isotype	37.4±0.2	1.7±0.1	1792.9±49.0	1792.9±49.2
Anti-GPIbα	37.2±0.6	1.4±0.1	1454.0±32.7	1999.8±68.7
Anti-Ly6G	38.6±0.7	1.4±0.1	1386.0±61.3	1954.3±52.1
Isotype	38.4±0.4	1.6±0.1	1601.9±66.1	1930.7±25.9
Anti-GPIbα	38.2±0.3	1.6±0.1	1633.1±29.1	1965.6±52.2
Anti-Ly6G	38.4±0.2	1.4±0.1	1426.2±54.5	1919.3±38.9
Isotype	36.0±0.4	1.4±0.1	1504.3±89.7	1985.5±10.3
Anti-GPIbα Fab‘	35.8±0.3	1.4±0.1	1499.6±96.9	2213.8±292.0
Vehicle	35.5±0.4	1.2±0.1	1388.9±105.1	1989.3±43.5

GR144053	36.7±0.4	1.2±0.1	1230.3±117.4	2030.6±48.5
Isotype	35.7±0.6	1.2±0.1	1299.2±42.2	1917.30±18.8
Anti-VWF	36.3±0.6	1.6±0.1	1710.9±103.5	1939.7±76.5
Isotype	37.8±1.0	1.3±0.2	1310.9±246.7	2018.6±114.7
Anti-CD40	42.0±0.6	1.3±0.1	1204.0±45.0	1990.9±16.6
Anti-CD40L	41.7±1.0	1.1±0.1	1036.0±33.8	1984.2±10.6
Anti-CD62P	37.3±0.7	1.4±0.1	1233.5±119.0	2037.1±42.5
Anti-CD11b	35.8±0.2	1.1±0.1	1210.2±35.6	1896.3±75.3
Vehicle	36.2±0.5	1.4±0.1	1458.6±64.4	1956.0±45.8
DNAse I	36.3±0.1	1.4±0.1	1478.7±7.4	1954.2±25.9

Supplemental videos

Video S1. Microvascular thrombus formation upon photochemical injury. Using fluorescence *in vivo* microscopy on the cremaster muscle of WT mice, thrombus formation elicited by photochemical injury was visualized as detailed in *Methods*, a representative video of a postcapillary venule is shown. Arrows point to the sites of first platelet adherence. The photochemical injury begins with the onset of the video and was applied to the entire field of view.

Video S2. Spatio-temporal dynamics of platelet aggregation during microvascular thrombus formation under unstimulated conditions. Using multi-channel *in vivo* fluorescence microscopy on the cremaster muscle of WT mice, thrombus formation elicited by photochemical injury was visualized as detailed in *Methods*. A representative video demonstrating the spatio-temporal aggregation dynamics of fluorescence-labeled platelets (GPIbβ derivate, X-649; DyLight) during thrombus formation in capillary and venular microvessel segments under unstimulated conditions is shown. The photochemical injury begins with the onset of the video and was applied to the entire field of view.

Video S3. Spatio-temporal dynamics of platelet aggregation during microvascular thrombus formation at the primary site of inflammation. Using multi-channel *in vivo* fluorescence microscopy on the inflamed cremaster muscle of WT mice, thrombus formation elicited by photochemical injury was visualized as detailed in *Methods*. A representative video demonstrating the spatio-temporal aggregation dynamics of fluorescence-labeled platelets (GPIbβ derivate, X-649; DyLight) during thrombus formation in capillary and venular microvessel segments in inflamed tissue is shown. The photochemical injury begins with the onset of the video and was applied to the entire field of view.

Video S4. Discoid platelets during microvascular thrombus formation. Using fluorescence *in vivo* microscopy on the cremaster muscle of WT mice, thrombus formation elicited by photochemical injury was visualized as detailed in *Methods*, a representative video of a postcapillary venule is shown. Arrows point to discoid platelets. The photochemical injury begins with the onset of the video and was applied to the entire field of view.

Video S5. Interactions of platelets and neutrophils during microvascular thrombus formation at the primary site of inflammation. Using multi-channel *in vivo* fluorescence microscopy on the inflamed cremaster muscle of WT mice, thrombus formation elicited by photochemical injury was visualized as detailed in *Methods*. A representative video demonstrating the spatio-temporal interaction dynamics of fluorescence-labeled platelets (white; GPIbβ derivate, X-649; DyLight) and neutrophils (orange; anti-Ly6G Phycoerythrin) during thrombus formation in capillary and venular microvessel segments in inflamed tissue is shown.

Video S6. Spatio-temporal dynamics of platelet aggregation during microvascular thrombus formation at the primary site of inflammation in neutrophil depleted mice. Using multi-channel *in vivo* fluorescence microscopy on the inflamed cremaster muscle of neutrophil-depleted WT mice, thrombus formation elicited by photochemical injury was visualized as detailed in *Methods*. A representative video demonstrating the spatio-temporal aggregation dynamics of fluorescence-labeled platelets (GPIbβ derivate, X-649; DyLight) during thrombus formation in capillary and venular microvessel segments in inflamed tissue is shown. The photochemical injury begins with the onset of the video and was applied to the entire field of view.

Video S7. Platelet interactions in microfluidic devices. Interactions of fluorescence-labeled platelets (GPIbβ derivate, X-649; DyLight) with bump structures were analyzed *in vitro* in custom-made PDMS microfluidic devices perfused with mouse blood by high-resolution microscopy as detailed in *Methods*, a representative video is shown.
